# COVID-19 vaccine hesitancy and patient self-advocacy: a statistical analysis of those who can and can’t get vaccinated

**DOI:** 10.1186/s12889-022-13661-4

**Published:** 2022-07-05

**Authors:** Douglas Ashwell, Joanna Cullinane, Stephen M. Croucher

**Affiliations:** 1 School of Communication, Journalism and Marketing, Massey University, Wellington, New Zealand; 2 Massey Business School, Massey University, Palmerston North, New Zealand

**Keywords:** Vaccine hesitancy, COVID-19, Patient self-advocacy, Health communication, New Zealand vaccine response, Vaccine literacy

## Abstract

**Background:**

This study applies the Patient Self-Advocacy scale to investigate vaccine hesitancy in New Zealand. Due to New Zealand’s very limited tertiary hospital system and vulnerable populations, the Government’s strategy to address COVID-19 has been to prevent the virus from entering the nation and to eliminate it when it does cross the border. Therefore, there is no opportunity for the nation to generate any acquired immunity through exposure. To transition from closed borders, New Zealand will need to run a highly successful national vaccination programme and this needs to have the ability to drive influential public health messaging to the targeted places within the communities where vaccine hesitancy most exists.

**Methods:**

This study employed statistical methods. A nationally representative survey of adults in New Zealand (*n* = 1852) was collected via Qualtrics. Independent samples t-tests, and multiple regression were used to explore the research questions.

**Results:**

Those who identify as medically able to be vaccinated expressed significantly higher confidence in the COVID-19 vaccine than those who identified as unable to be vaccinated. Patient-self advocacy had a positive effect on vaccine confidence. Individuals who identify as able to be vaccinated have less hesitancy. Demographics had various effects on vaccine hesitancy.

**Conclusion:**

The research highlights particularly important insights into vaccine hesitancy related to patient self-advocacy behaviours, and various demographic variables such as political affiliation. In addition, the research adds further clarity on how and why New Zealanders have responded to the COVID-vaccine. Finally, the importance of vaccine literacy is discussed.

The COVID-19 virus reached pandemic status in March 2020. Since then, the virus has spread to more than 213 nations and territories, infected more than 186 million people, and resulted in more than 6 million deaths worldwide [1]. As the world grappled with the political, social, economic, and human effects of the virus, a number of vaccines were trialled and developed to counter the virus. Within a year of the outbreak, numerous global companies successfully managed to negotiate clinical trials and received approval for vaccines [2]. In mid-2021, vaccines began being administered throughout the world at varying rates to the public. In the lead up to, and then during the vaccine delivery, vaccine hesitancy/scepticism remains a consistent challenge [3, 4]. for example in an analysis of 19 nations found COVID-19 vaccine acceptance rates varied from 55% in Russia, 90% in China and that 41% of Austrians are hesitant toward COVID-19 vaccines [5, 6].

With research linking vaccine hesitancy with virus containment and infection rates [7] this study analyses vaccine hesitancy in New Zealand. At the time of writing New Zealand has had relatively few COVID-19 infections, deaths, and a largely successful government strategy to prevent the virus from entering the nation [8].

The current study explored vaccine hesitancy in New Zealand. In particular, we conducted a nationally representative online survey among New Zealanders who stated they can or cannot get vaccinated. To fully examine vaccine hesitancy, we measured demographic variables, vaccine hesitancy, and patient self-advocacy [9, 10].

## Background

### Vaccine hesitancy and attitudes about vaccines

Public health vaccination programmes in many countries have resulted in reductions of mortality and morbidity associated with infectious diseases. Immunisation programmes have been assumed to have “all-but” defeated poliomyelitis, smallpox, and tuberculous (prior to later resurgences). However, fundamental to vaccination programmes being successful is the uptake level among the targeted population. This is because no vaccine has complete efficacy and in addition to the direct protection afforded to individuals who acquire immunity from the vaccination, it is believed those people in turn act as a shield for others in the population who are either unvaccinated or who have not acquired immunity from the vaccination i.e. protection through ‘herd immunity’ [11].

An important dimension related to public health vaccination programmes has been the scepticism or reluctance expressed towards vaccines and the attendant risks associated with insufficient uptake jeopardising the public good of herd immunity and placing at risk those in the population who cannot receive the vaccination directly themselves because of other health conditions [11]. This reluctance or scepticism has been labelled “vaccine hesitancy”. This hesitancy is not a new phenomenon [12], and dissent about vaccination/inoculation has been expressed since vaccines were first developed [11, 13].

In 2019 the World Health Organisation (WHO) labelled vaccine hesitancy as one of its top 10 threats to global health [14]. The WHO’s Strategic Advisory Group of Experts (SAGE) Working Group on Vaccine Hesitancy defines vaccine hesitancy as the refusal or delay in accepting vaccines despite vaccination services being readily available [15]. The SAGE definition of vaccine hesitancy is one term among many, including: “vaccine sceptical”, “vaccine refusers”, “anti-vaccinationist”, and “anti-vaxxers” used to describe those who are unwilling to vaccinate. The latter term is often used in a pejorative manner describing those who are considered under-informed or “crazy” who have inexplicable beliefs that are counter to scientific evidence [16]. Whatever label is used, it must be understood that vaccine hesitancy describes a heterogenous group who have varying views on vaccination. Some may refuse to vaccinate while others may choose some vaccines, refuse others, delay vaccination or vaccinate despite having misgivings about doing so [17].

Reasons for some people to refuse vaccines are varied, some motivations are religious in nature [16], others’ non-compliance with vaccine programmes is underpinned by an individualist epistemology i.e. a philosophical view that the individual is sovereign in their own decision making and must determine their own “truth” [16]. A further explanatory factor in vaccine hesitancy are alternative views (non-scientifically attested) of how the immune system works. For example, the philosophically anthroposophic and those who believe in the supremacy of acquiring natural immunity and those who believe in complementary and alternative medicines, are all likely to hold hesitant, reluctant, or rejecting views towards immunisations [16]. These beliefs are sometimes labelled salutogenic [18]. People holding such beliefs view the immune system as central to the body’s overall health and therefore believe it must be protected or trained so it can function effectively against the many challenges posed by the environment [19]. Those believing in natural immunity believe vaccination actually threatens the effectiveness of the immune system. Furthermore, some individuals believe disease strengthens children mentally and physically, with inflammations and fevers associated with the diseases seen as a way for the body to ‘cleanse’ itself [20].

A number of studies have also illustrated that vaccine hesitancy also occurs as a result of individual’s poor experiences with the health system or health professionals [21]. This can especially be the case for ethnic minorities who often suffer racial discrimination or have information supplied in a culturally inappropriate manner [22, 23].

Another reason for vaccine hesitancy is concern about the safety of vaccines. In 1955, a manufacturing flaw with the polio vaccine resulted in 250 people catching polio and cross infection of the vaccines with Simian Virus SV40 (1955–1963) resulted in significant decreases in the proportion of people accepting the polio vaccine [24]. In 1976, it was found that a small number of people who received the swine flu vaccine contracted Guillain–Barre Syndrome and as the causative link has been unable to be proven, that vaccine was withdrawn [24]. In 1982, the U.S.’s NBC network broadcast the investigative programme: *DPT: Vaccine Roulette.* The programme reported the widely administered childhood vaccine against diphtheria, pertussis,^1^ and tetanus was linked to encephalitis among children. The reaction was a widespread refusal of that vaccine among parents in the U.S. and the revision of diphtheria, pertussis, and tetanus from rare chronic conditions when the vaccine programmes were in place, to a return to epidemic levels of the diseases. Similarly, in 1998 Andrew Wakefield (then a medical doctor) published an article in the *Lancet*suggesting a link between the measles, mumps and rubella (MMR) vaccine and autism in children. The paper caused a rejection of the vaccine by many parents and this has persisted even though the paper was later debunked and retracted for flaws in the research and for failure by Wakefield to declare material conflicts of interest. Wakefield and his research team were also later accused of research fraud. Similarly, there were concerns of a link between the hepatitis B vaccine and multiple sclerosis in France in 1998, which were later determined to be unfounded, but awareness of these concerns still impacts uptake of that vaccine in France [24]. Social media also highlights many cases of vaccines that were knowingly mis-sold or misrepresented or counterfeited e.g. in China, Indonesia, the Philippines, and India [25].

These incidents and themes of distrust are rapidly spread by the Internet and social media. People seeking health information on the Internet is now widespread. For example, 72% of U.S. respondents reported they sought health information on the Internet [26]. A survey in Norway found 62% of adults used the Internet to search for health information [27]. These examples are part of the move toward individuals self-advocating for their own health care.

### Patient self-advocacy

This increased involvement includes asking more questions of providers, seeking out more information, and verifying information about treatments and medical decisions [28, 29]. Being more involved in one’s health care is defined as self-advocacy [10]. When individuals are faced with a health issue, the issue produces a self-help response where individuals become advocates for their own treatment/care. Three dimensions of patient self-advocacy have been identified: increased illness and treatment education, increased assertiveness in health care interactions, and increased potential for nonadherence [10].

Many individuals want information about their illness. Individuals who are actively interested or engaged in their medical care are likely to seek out more information about their condition or medical treatment(s), as information provides an outlet for individuals to assert themselves in the patient-provider interaction [10]. However in a countervailing trend, health misinformation is rampant, particularly online. Health misinformation has been defined as health-related fact claims that are currently false due to a lack of current scientific evidence [30]. As more and more individuals seek out information about vaccines online and via social media, it is difficult to ascertain the validity of the information individuals are consuming about the efficacy of vaccines [31].

The second dimension of patient self-advocacy is assertiveness in health care interactions. In health interactions, particularly ones that are uncertain, individuals will often have an increased need for autonomy [10]. This need for autonomy will lead many to ask more questions of providers, and to be more sceptical. For example, research has found when health care providers themselves are patients they are more likely to get vaccinated when they are not *told*they have to be vaccinated [32]. Demands or required vaccinations are likely to diminish trust in vaccinations and adhering to vaccination policies. Furthermore, when individuals feel pressured into getting vaccinated, the vaccines lack moral justification and thus the individuals are less likely to get vaccinated [33]. Overall, researchers have shown that when individuals feel they have more autonomy and can interact more with their health care providers in making a vaccine choice, intent to vaccinate is higher [32, 34].

The third dimension of self-advocacy is mindful nonadherence. This is the tendency of individuals to reject treatments or to not follow treatments when they do not meet expectations [10]. Adherence to provider recommendations is critical to positive health outcomes. Research shows communication between patients and providers is integral in determining if patients adhere to provider’s orders [35]. A variety of choices influence whether or not a patient will adhere to a provider’s orders, particularly in terms of getting a vaccination. Factors such as cost of a vaccine, transport costs to a facility, distance to the clinic/facility, previous negative medical experiences, lower educational level, lower socio-economic status, and minority racial/cultural status have all been found to increase vaccine hesitancy [36, 37].

The current study utilises the Patient Self-Advocacy Scale (PSAS) to examine vaccine hesitancy in New Zealand taking into account whether or not a person believed they could receive a vaccine. To date there have been few studies of vaccine hesitancy utilising the Patient Self-Advocacy Scale. However, a recent study of Australian parental attitudes to vaccine used the scale [38]. The study identified 5 groups of parents: 1) those who believed in all childhood vaccines with no concerns, 2) those who believed in all vaccinations with some concerns, 3) parents who believed in most but not all childhood vaccines, 4) those who believed in some childhood vaccines and 5) those who believed in no childhood vaccines. The study found parents in the first two groups had lower education, and non-adherence scores on the PSAS in comparison to those in the (most) group and those in group 1 (all unconcerned) also had low self-assertiveness scores on the scale in comparison to the other groups [38].

### Research questions

New Zealand has had relatively few COVID-19 cases, 22,330 cases and 53 deaths as of February 2022 [39]. A combination of contact tracing, stringent border control initiatives, and effective health communication campaigning has limited the spread of the virus thus far [40]. As the nation launched its vaccine rollout in mid-2021, questions over the speed of the vaccine delivery, efficacy of the vaccines, questions over who is medically exempt or unable to receive vaccinations, and concerns over misinformation increased [41–43]. Nearly 1 in 3 New Zealanders remain hesitant or sceptical about COVID-19 vaccines. Much of this hesitancy is linked to misinformation individuals receive about vaccines and low levels of trust in medical professionals [44]. In addition, misinformation among many as to whether they are able to receive vaccinations, coupled with some health practitioners themselves spreading misinformation about the COVID-19 vaccination [41], and blame over the spread of the COVID-19 virus has heightened hesitancy [45].

The New Zealand government has devoted significant attention to increasing vaccine intention by educating its population as to who can get vaccinated, the effects of the vaccine, and encouraging people to consult with their medical providers. Additionally, the national campaign “Unite Against COVID-19” has stressed how the vaccine is free of charge, how New Zealanders will be able to get vaccinated at any local provider, and how it is critical for all New Zealanders to get vaccinated to protect their family or whānau (a Māori-language word for extended family) [46].

In New Zealand, like in many other nations, there is still significant misunderstanding, misinformation, and hesitancy toward the COVID-19 vaccination and vaccines in general. Moreover, as different vaccines have different components, and respond to the body differently, the Centers for Disease Control and Prevention (CDC), and other similar international associations have advised particular individuals to avoid receiving specific vaccines [47]. Advice on the COVID-19 vaccines is still not clear from the CDC and similar associations. To first understand New Zealanders’ differing understanding of who can and cannot receive the COVID-19 vaccination, we propose the following:

- RQ1: To what extent do New Zealanders identify as able to receive a COVID-19 vaccine?
  To examine vaccine hesitancy, we explored the extent to which New Zealanders have hesitancy toward the COVID-19 vaccine. We investigated the link between patient self-advocacy, and vaccine hesitancy among those who identify as being able to and unable to receive the COVID-19 vaccine:
- RQ2: To what extent does vaccine hesitancy differ among those who identify as able and unable to receive a COVID-19 vaccination?
- RQ3: To what extent does patient self-advocacy predict vaccine hesitancy?

## Methods

After ethical approval, a nationally representative sample survey of New Zealand adults was collected in April–May 2021. The survey was conducted by Qualtrics, a survey agency. Qualtrics panels are comparable to other populations in published research [48]. Participants for this study included 1852 New Zealanders, a response rate of 35% of individuals contacted. Participants completed the Vaccine Hesitancy Scale [9], the Patient Advocacy Scale [10], demographic questions, a question asking if they are able to receive the COVID-19 vaccination, and a follow-up question (if unable to receive the vaccination) asking them why they are unable to receive the vaccination. Table 1 presents the full demographic information for all participants. Confirmatory factor analyses (CFAs) were conducted on all measures. Established criteria were followed [49]. See Table 2 for means, standard deviations, and alphas associated with the study variables.

**Table 1:** Demographic information

Variable	*n*	*M*	*SD*
Age		42.41	16.79
Can you get vaccinated?			
Yes	1412 (76.2%)		
No	440 (23.8%)		
Why Can’t you get vaccinated?			
Guillian-Barre Syndrome	3 (.7%)		
Liver Disease	61 (13.9%)		
Asthma	78 (17.7%)		
Kidney Disease	33 (7.5%)		
Lyme Disease	18 (4.1%)		
Pregnant or Trying to be	106 (24.1%)		
Doctor Said I can’t	21 (4.8%)		
Religious Leader said I can’t	41 (9.3%)		
Miscellaneous Cancer	30 (6.8%)		
Didn’t Provide Reason	49 (11.1%)		
Sex			
Male	830 (44.8%)		
Female	1021 (55.1)		
Other	1 (.1%)		
Do you Have Private Health Insurance			
Yes	632 (34.1%)		
No	1220 (65.9%		
Race			
Pākehā (White)	926 (50%)		
Māori	557 (30.1%)		
Pacific/Islander	224 (12.1%)		
Other	145 (7.8%)		
Highest Educational Level			
High School	719 (38.8%)		
2-year degree/University	313 (16.9%)		
University degree	706 (38.1%)		
Post Graduate Degree	114 (6.1%)		
Political Affiliation			
National	487 (26.3%)		
Labour	797 (43%)		
Green	115 (6.2%)		
Māori	37 (2%)		
Other	59 (3.2%)		
No Affiliation	357 (19.3%)

**Table 2:** Means, standard deviations, reliability coefficients, and correlations

	Combined Sample *n* = 1852						
Variable	*M*	*SD*	*α*	(1)	(2)	(3)	(4)
(1) Confidence in Vaccines	4.90	1.94	.97	-			
(2) Info Seeking	2.99	.85	.78	.21**	-		
(3) Assertiveness	2.87	.75	.74	.11**	.67**	-	
(4) Mindful Noncompliance	2.84	.90	.86	-.13**	.39**	.52**	-
	Not able to Vaccinate Sample *n* = 440						
Variable	*M*	*SD*	*α*	(1)	(2)	(3)	(4)
(1) Confidence in Vaccines	2.31	1.50	-	-			
(2) Info Seeking	2.59	.94	-	.15**	-		
(3) Assertiveness	2.58	.89	-	.18**	.71**	-	
(4) Mindful Noncompliance	2.69	.96	-	.06	.45**	.58**	-
	Able to Vaccinate Sample *n* = 1412						
Variable	*M*	*SD*	*α*	(1)	(2)	(3)	(4)
(1) Confidence in Vaccines	5.71	1.22	-	-			
(2) Info Seeking	3.12	.78	-	-.05	-		
(3) Assertiveness	2.96	.68	-	-.20**	.62**	-	
(4) Mindful Noncompliance	2.89	.88	-	-.45**	.35**	.49**	-

Note: ** p < *.*001

### Vaccine Hesitancy Scale

The 14-item Vaccine Hesitancy Scale (VHS) was adopted to assess vaccine hesitancy [9]. The VHS is a 5-point Likert-type scale ranging from s*trongly disagree* to *strongly agree.*The VHS has two factors: confidence and risk perception. Sample items include, “Vaccines are effective,” “New vaccines carry more risks than older vaccines,” “I feel comfortable getting vaccinated,” and “Governments over hype the need for vaccines.” Reliabilities have ranged from 0.80 to 0.95 [9]. In this study the two factor-solution did not fit the data: χ2(19) = 684.03, *p* < 0.0001, CFI = 0.88, RMSEA = 0.15, GFI = 0.92. However, a one-factor solution with confidence in vaccines was a valid solution: χ2(9) = 597.62, *p* < 0.001, CFI = 0.96, GFI = 0.95, SRMR = 0.05. Thus, a higher score on VHS indicates higher confidence in vaccines. Therefore, for the purpose of data interpretation, the variable “vaccine hesitancy” will be labelled “vaccine confidence”.

### Patient Self-Advocacy Scale

The 18 item Patient Self Advocacy scale was adopted [10]. This scale assesses patient self-advocacy across three dimensions: increased illness and treatment education, increased assertiveness in health care interactions, and increased potential for nonadherence. The scale is on a 5-point Likert-type scale ranging from *strongly disagree* to *strongly agree.*Sample items include: “Sometimes there are good reasons not to follow the advice of a physician,” and “I actively seek out information on my illness.” Reliabilities for the measure have ranged from 0.70 to 0.92 [10, 50]. CFA confirmed a three-factor solution after deleting five items (1, 2, 7, 10, and 17): χ2(62) = 909.36, *p* < 0.001, CFI = 0.95, GFI = 0.95, RMSEA = 0.05.

### Statistical analysis

To address the research questions, various statistical approaches were employed. To answer RQ1, a simple frequency analysis was conducted. To answer RQ2, an independent samples *t*-test was conducted comparing those who identify as able and unable to receive a COVID-19 vaccination. To answer RQ3, multiple regression was used. Vaccine confidence was entered as the predictor variable. The following predictor variables were entered: ability to get a vaccine, age, sex, race, highest educational level, political affiliation, does the participant have private medical insurance, and a dimension of patient self-advocacy (Info Seeking, Assertiveness, and Mindful Noncompliance). Dummy variables were created for race and political affiliation, with Pākehā and the Labour Party serving as reference groups. Cross-product terms were created to test for interaction effects.

### Results

#### Ability to get vaccinated

The first research explored the extent to which New Zealanders identify as able to receive a COVID-19 vaccine. Of the 1852 participants, 440 identified as not being able to receive a COVID-19 vaccination, 23.75%. Of those particiants, individuals who identified themselves as having Guillain-Barré Syndrome (GBS), Liver disease, kidney disease, and cancer meet the criteria set by groups such as the CDC (28.9% of the 23.75% of unable to receive vaccine participants). Those individuals who stated they could not receive the vaccination due to asthma, Lyme Disease, pregnancy or trying to get pregnant, and/or doctor said I can’t made up 50.7% of the unable to receive vaccination group. These reasons fall within a grey area as per CDC and other association guidelines [47].

### Vaccine hesitancy

Independent samples *t*-test compared those who identify as able and unable to receive a COVID-19 vaccination on vaccine hesitancy (RQ2). Those who identify as medically able to be vaccinated expressed significantly higher confidence (*M* = 5.71, *SD* = 1.22) in the vaccine than those who identified as unable to be vaccinated (*M* = 2.31, *SD* = 1.50); *t*(1850) = -48.09, *p* < 0.001.

To answer RQ3, multiple regression was used. In model 1, ability to get a vaccine was entered (*R*^2^= 0.55). In model 2, age, sex, race, educational level, political affiliation, and if a participant has private medical insurance were entered. This model was a significant improvement over model 1 (*R*^2^= 0.60; *ΔF* = 16.45, *p* < 0.001). In model 3, Info Seeking, Assertiveness, and Mindful Noncompliance (patient self-advocacy) were entered. This model was a significant improvement over model 2 (*R*^2^= 0.64; *ΔF* = 71.41, *p* < 0.001). In model 4, cross-products of ability to get vaccinated and patient self-advocacy were entered to determine the effects of ability to get a vaccination and patient self-advocacy on vaccine confidence/hesitancy. This model was a significant improvement over model 3 (*R*^2^= 0.66; *ΔF* = 29.21, *p* < 0.001), suggesting a significant interaction effect. Model 4 was retained for final analysis. Regression results are presented in Table 3.

**Table 3:** Regression model for confidence in COVID-19 vaccine

Regressor	Model 1		Model 2		Model 3		Model 4	
	*b*	*SE*	*b*	*SE*	*b*	*SE*	*b*	*SE*
Intercept	-1.08	.13	-.96	.21	-.33	.22	-3.42	.44
Vaccine Possible	.75**	.07	.64**	.09	.65**	.09	1.07**	.25
Age			.19**	.01	.16**	.01	.15**	.01
Female			.04*	.06	.05*	.06	.05**	.06
Māori			-.01	.07	-.01	.07	-.02	.06
Pacific/Islander			-.01	.10	-.01	.09	-.01	.09
Other Race			.02	.11	.01	.11	.01	.10
National Party			-.08**	.08	-.07**	.07	-.08**	.07
Green Party			.02	.13	.04*	.12	.03	.12
Māori Party			.01	.21	.01	.20	.01	.19
Other Political Party.01			.21	.01	.16	.01	.16	
No Political Affiliation			.03	.08	.03*	.08	.03*	.07
Private Insurance-.09**			.06	-.09**	.06	-.08*	.06	
Highest Educational Level			.06**	.03	.06**	.03	.05**	.03
Info Seeking					.10**	.04	-.03	.17
Assertiveness					.01	.05	.26**	.20
Mindful Noncompliance					-.23**	.04	.15*	.15
Info Seeking*Vaccine Possible							.18	.10
Assertiveness*Vaccine Possible							-.38**	.11
Mindful Noncompliance*Vaccine Possible							-.50**	.08
*F*	2312.89***		210.93***		204.47**		184.74**	
*ΔF*			16.45**		71.41**		29.21**	
*R*^2^	.55		.60		.64		.66	
*R*^2^_*adj*_	.55		.60		.64		.65	

Note: * p < .01, ** p < *.*001

As model 4 in Table 3 depicts, there are multiple main and interaction effects in predicting vaccine confidence. Assertiveness (*b* = 0.26) and Mindful non-compliance (*b* = 0.15) both have positive effects on confidence. Individuals who identify as being able to be vaccinated tend be more confident, and thus have less hesitancy (*b* = 1.07). Age (*b* = 0.15) and educational level (*b* = 0.05) have positive effects on vaccine confidence. Women were higher in confidence (*b* = 0.05) than men. Regarding political affiliation, individuals who did not identify with a political party had higher levels of confidence (*b* = 0.03), while National voters (*b* = -0.08) were lower in confidence when compared to Labour party voters. Not having private insurance had a negative effect on vaccine confidence (*b* = -0.08).

Regarding the interaction effects, the following results were found. First, assertiveness among individuals who are able to be vaccinated had less of an effect on vaccine confidence compared to those who are unable to be vaccinated (*b* = -0.38). Assertiveness is seen as a demand for autonomy in health care decisions. Therefore, those individuals who are able to be vaccinated are less likely to demand autonomy or to assert independence in vaccine decisions. Similarly, mindful noncompliance among individuals who are able to be vaccinated also had less of a negative effect on vaccine confidence (*b* = -0.50) compared to those who are unable to be vaccinated. The tendency to reject medical treatments and not-comply is not as strong among the group able to receive vaccines.

## Discussion and conclusion

Research suggests for New Zealand to have any chance of achieving herd immunity against new COVID-19 variants, in particular the delta or omicron variants, vaccination rates will need to be well over 90% [51]. Therefore, it is imperative to understand the factors that influence peoples’ willingness to get vaccinated. The current study adds to the knowledge of vaccine hesitancy by understanding the reasons given for not being able to have a vaccine and also the differing vaccine attitudes between those who report being able to have vaccines and those who do not.

Our model predicts up to 65% of COVID-19 vaccine hesitancy. Those who were more confident or had higher levels of self-efficacy were more likely to be willing to receive COVID-19 vaccines and this is similar to previous research into vaccine uptake [52]. Women were more confident than men in New Zealand, but interestingly, this result differs from recent research which has found men are more confident than women in relation to COVID-19 vaccines [53, 54]. These differing results could be attributed to the nature and form of the New Zealand government’s vaccine rollout campaign, the centralised messaging during the pandemic, and/or a variety of other health messaging related issues. More research should explore why women in New Zealand are more confident than men in vaccines.

The decreased confidence in COVID-19 vaccines among those identifying as National party voters, a conservative party, is similar to overseas research finding conservatives were less likely to intend to vaccinate [55]. While not directly related to COVID-19 vaccines, research has found political conservatism related to a lower level of trust in science, which indirectly and negatively affected willingness to comply with COVID-19 safety measures [56]. Whether political conservatives in New Zealand follow the same trend is an area for further research.

Nearly 25% of those surveyed for this study, reported they were unable to receive a vaccine. However, of that 25% of the total sample, nearly 75% of this group did not meet criteria considered legitimate by the CDC for not receiving a vaccination. This group either fell into a medically grey area, gave religious reasons, or no reason at all. It is this latter group that is of concern if New Zealand or other countries are to achieve herd immunity. The biggest group in the medically grey area were women who identified themselves as pregnant or trying to become pregnant (24.1%) of the 25% who said they could not get vaccinated. A number of previous studies have found pregnant women are more likely to be vaccine hesitant [57]. Given previous research showing the reactions of pregnant women to vaccines, it appears to follow that women trying to get pregnant may be concerned that vaccines harm their chances of becoming pregnant. However, all advice from the New Zealand Ministry of Health (MoH) and the CDC state women who are pregnant, trying to get pregnant and breastfeeding can safely have the COVID-19 vaccine [47, 58]. The next largest group in the medical grey area stated they could not receive a vaccine because they had asthma (17.7%). Given COVID-19 has serious effects on the respiratory system it is argued this would be a group being encouraged to receive the vaccine. Indeed the New Zealand Government’s Unite against COVID-19 website, encourages all those who have underlying health conditions, including asthma, to get a COVID-19 vaccine [59]. These results concerning pregnancy and asthma suggest MoH messaging is not being clearly received by some of those eligible to receive the COVID-19 vaccine. This is of concern as women who contract COVID-19 during pregnancy and those with asthma can become seriously ill. More targeted communication may be needed for these two groups concerning the benefits of and their ability to have COVID-19 vaccines. Previous research has illustrated, in addition to public health campaigns, insuring that frontline medical staff, physicians, midwives and nurses, have factually based responses to frequently asked questions or misinformation about the COVID-19 and other vaccines, can alleviate fears among women who are pregnant or wishing to become pregnant [57, 60, 61]. Such information may also be able to be used to alleviate the fears of those with conditions such as asthma [57]. Therefore, in addition to the current public health messaging, New Zealand should invest in training frontline medical staff to insure they have the required information so they can feel confident when confronted with concerns and objections.

For the last group in the medically grey area, those identifying with Lymes disease (4.1%), the picture is less clear. There is no clear consensus on if Lyme disease sufferers are unable to get vaccinations [47]. Many of the symptoms of Lyme disease and long-haul COVID-19 are similar [62] and so more medical research may be required to definitively answer whether or not Lyme disease sufferers can received COVID-19 vaccines.

Just over two percent of those surveyed stated they could not get a COVID-19 vaccine because their religious leader told them not to get a vaccine. This percentage is similar to the U.S. where those seeking non-medical exemptions rose from 1.48% to 2.2% between 2004–2011 [63]. While this does not seem to be a high figure overall it does not take into account that those who seek such exemptions seem to cluster geographically. Such geographic clustering also occurs in New Zealand with the West Coast District Health Board suggesting several religious groups who did not believe in immunisation meant the Board would never reach its immunisation targets [64]. This means these areas may remain susceptible to COVID-19 and could be a source of infection for the rest of the country unless properly managed. Religious objections to vaccines have been present since the first vaccines were produced [13]. However, ‘religious reasons’ to refuse vaccines are often not theologically based, rather they reflect the safety concerns of a faith based social network of people [65]. Therefore, further research in the New Zealand context may be beneficial to investigate whether religious reasons arise from inherently close social networks or from particular beliefs. In some cases these religious objections may be overcome by frontline medical staff pointing out that high profile religious leaders like the Pope have encouraged vaccination.^58.^ These high profile messages could also be repeated in public health campaigns.

Of more concern are those that did not provide a reason for their inability to have a vaccine. Without an understanding of this group’s inability to receive a vaccine it is impossible to construct any meaningful communication strategies to deal with any concerns this group might have about Covid-19 vaccines. This is an area for more in-depth research.

Health communication research demonstrates that patients who are more involved in their treatments and medical decisions tend to seek out more information, evaluate treatments based on their effectiveness and communicate with providers with more assertiveness [66, 67]. The results of the current study reveal that seeking out information, even if one is “unable” to get a vaccine, is linked with increased confidence in vaccines. This link demonstrates the importance of information provision in reducing vaccine hesitancy. As individuals seek out information, their confidence usually increases, decreasing hesitancy. In addition, the need for autonomy (assertiveness) increases hesitancy in those “unable” to be vaccinated. For those able to be vaccinated, the need for autonomy and mindful non-compliance both decrease confidence in vaccines/increased hesitancy. These results demonstrate the importance of considering patient self-advocacy behaviours when examining vaccine hesitancy. As the results show, an individual’s level of involvement in their own treatment or medical decision-making has a significant impact on their level of hesitancy or confidence in vaccines.

The results of the current study underscore the importance of health and vaccine literacy which are essential to permit patients to both obtain and evaluate health/vaccine information, and to respond to (mis)information about vaccines. Thus, it is critical for governments to develop informative campaigns that confront public concerns directly [68].

While this study is a national sample of the New Zealand public, the study is cross-sectional in its design. Thus, the study is not able to measure vaccine hesitancy, confidence, nor patient self-advocacy longitudinally. The data for this study were collected in April–May 2021, and it is highly likely that attitudes toward vaccines will shift as the vaccine rollout intensifies in New Zealand. Future research should explore these trends longitudinally. Second, while the study was drawn from national databases held by Qualtrics, the sample is not fully representative of the New Zealand population. For example, less than 1% of this sample identified as an other sex/gender, while more than 1% of the population identify as such. Future research could further strive for an even more representative sample, Third, while the sample closely resembled the demographics of New Zealand, we did not track participant geographic location. New Zealand is a largely rural country of five million people. The country has one urban area of over 1 million people (Auckland) and two other areas of 200,000 + people (Christchurch and Wellington). Future research should examine vaccine hesitancy among rural versus urban dwellers, particularly as journalists and researchers in New Zealand have alluded to differences in the perceptions between the two groups on vaccines [69].

This research investigated vaccine hesitancy in New Zealand by examining the scale and causes of hesitancy and related behavioural characteristics. The research has generated a model that predicts up to 65 percent of COVID-19 vaccine hesitancy in New Zealand. There are three notable findings: first that many people incorrectly identify themselves as “unable” to receive the vaccine. Second*,* autonomy in health care choices increases hesitancy in those who identify themselves as “unable” to be vaccinated. Third, the behaviour of “seeking out information” about vaccines, is significantly linked with increased confidence in vaccines. Together, these findings are relevant in assisting the New Zealand Government in public health communication messages to reduce hesitancy as the vaccine roll-out intensifies. In particular, it would be beneficial for the New Zealand Government to redirect communication strategies towards addressing balanced evaluative information to the New Zealand public through trusted routes. In particular, the hesitancy of pregnant and hoping to become pregnant women is an area requiring a focused communication plan. Similarly communication to New Zealanders with health conditions such as asthma requires focused attention as this group are a sizeable minority who are both hesitant and at greater risk.

More specifically, communication strategies and health practitioner training which focus on generating patient self-advocacy, information seeking behaviour, and culturally literate engagements are medium to long-term strategies that will help with hesitancy issues. This investment in fundamental change to the practitioner-patient communication exchange seems justified given that COVID-19 will persist as an endemic disease requiring regular seasonal vaccination and also reflects the fact that even pre-COVID, public health strategies were increasingly blighted by vaccine hesitancy and patients’ non-adherent behaviours.

## Data Availability

The data that support the study conclusions are unavailable for public access because informed consent to share the data (beyond the research team) was not obtained from study participants, but de-identified data is available from the corresponding author on reasonable request.

## Footnotes

1 Whooping cough.
